# Scaffold-Based Gene Therapeutics for Osteochondral Tissue Engineering

**DOI:** 10.3389/fphar.2019.01534

**Published:** 2020-01-14

**Authors:** Xin Yan, You-Rong Chen, Yi-Fan Song, Meng Yang, Jing Ye, Gang Zhou, Jia-Kuo Yu

**Affiliations:** ^1^ Knee Surgery Department of the Institute of Sports Medicine, Peking University Third Hospital, Beijing, China; ^2^ Key Laboratory for Biomechanics and Mechanobiology of Ministry of Education, School of Biological Science and Medical Engineering, Beihang University, Beijing, China

**Keywords:** microRNAs, gene therapy, scaffold, tissue engineering, osteochondral regeneration

## Abstract

Significant progress in osteochondral tissue engineering has been made for biomaterials designed to deliver growth factors that promote tissue regeneration. However, due to diffusion characteristics of hydrogels, the accurate delivery of signaling molecules remains a challenge. In comparison to the direct delivery of growth factors, gene therapy can overcome these challenges by allowing the simultaneous delivery of growth factors and transcription factors, thereby enhancing the multifactorial processes of tissue formation. Scaffold-based gene therapy provides a promising approach for tissue engineering through transfecting cells to enhance the sustained expression of the protein of interest or through silencing target genes associated with bone and joint disease. Reports of the efficacy of gene therapy to regenerate bone/cartilage tissue regeneration are widespread, but reviews on osteochondral tissue engineering using scaffold-based gene therapy are sparse. Herein, we review the recent advances in gene therapy with a focus on tissue engineering scaffolds for osteochondral regeneration.

## Introduction

**Figure 1:**
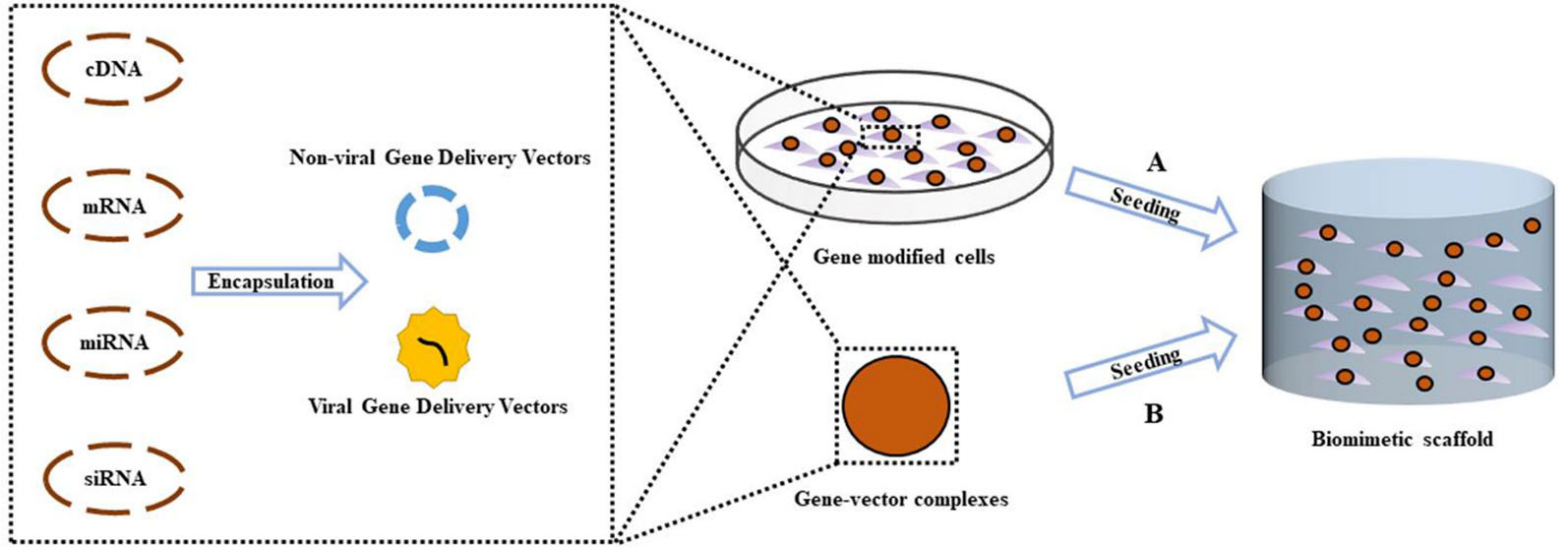
Scheme of gene activated scaffold. Specific gene sequences were encapsulated into gene delivery vectors (Non-viral or viral), forming gene-vector complexes. **(A)** Exogenous seed cells were modified by uptake of gene–vector complexes, then were seeded into biomimetic scaffold supporting for the formation of new tissue. **(B)** The gene–vector complexes were loaded directly into the scaffold. Endogenous seed cells around the osteochondral defect migrate into the scaffold and take in specific genes in the gene–vector complexes, promoting chondrogenic or osteogenic differentiation.

Articular osteochondral injury is a common and frequently occurring disease in orthopedics, mainly caused by accidental trauma, sports injury or arthritis. Mature articular cartilage has a very weak ability to resist injury and disease, and has limited self-repair ability. After the articular cartilage is damaged, it cannot be effectively repaired, eventually leading to the occurrence of osteoarthritis (OA). It is expected that by 2030, OA will be the most common chronic degenerative joint disease among aging populations (Thomas et al., 2014; Tsezou, 2014). OA patients often suffer from severe pain and limited mobility. OA is also considered the leading cause of disability in the general population. The regeneration of articular cartilage that lacks self-healing ability is a major challenge in clinical treatment and clinically available methods fail to meet long-term effective regeneration requirements. This has caused concern in the field of osteochondral tissue engineering in which new tissues can be engineered to promote joint regeneration and prevent the onset of OA (Zhang et al., 2019). One promising approach is the treatment of genes delivered by tissue engineering scaffolds. By transfecting specific gene sequences into seed cells, overexpressing or silencing the original gene, the biological function of the cells could be regulated to obtain the desired effect. Gene therapy combined with tissue engineering scaffolds provides a more precise, controlled, and sustained release of therapeutic factors compared to traditional methods of delivering growth factors directly (**Figure 1**). This review focuses on recent advances in gene therapy in the field of scaffold-based osteochondral tissue engineering. In terms of miRNAs, we focus on recent research progress related to OA in the hope that miRNA can be used in the future gene therapy approaches combined with scaffold-based osteochondral tissue engineering.

## Osteochondral Tissue Engineering

Tissue engineering uses bionic scaffold to simulate the cell growth microenvironment and combines the body's self-healing ability to guide tissue regeneration in damaged or defective tissue sites. The cell microenvironment of tissue engineering bionics can induce cartilage or the osteogenic differentiation of stem cells, promoting their proliferation and migration, leading to endogenous osteochondral regeneration (Li et al., 2016). Osteochondral tissue engineering has evolved to enhance cell proliferation, differentiation, migration, and survival by transmitting growth factors and signaling molecules. These ligands combined with cell surface receptors of mesenchymal stem cells or mesenchymal progenitors, activate signaling pathways that promote osteochondral regeneration. However, the short half-life of recombinant proteins, such as BMPs, low bioactivity and high preparation costs lead to the exploration of new methods to deliver bioactive osteochondral regenerative compounds (Shi et al., 2014). A promising area is the use of scaffold-based gene therapy. By introducing specific gene sequences into cells, it is possible to modify or replace existing genes and regulate their epigenetic functions to achieve a desired purpose (Ginn et al., 2018). Gene-activated scaffolds provide a continuously controlled method of nucleic acid therapy to achieve a more efficient and safe release of biological agents.

### Scaffold Biomaterials

To promote tissue regeneration, the osteochondral scaffold must be biocompatible, have a suitable rate of degradation, and possess a porous structure (Wang et al., 2019a). To-date, osteochondral tissue engineering biomaterials include natural polymers, synthetic polymers, metals, and inorganic materials. Natural materials are derived from animals, plants, and microorganisms and can be classified into protein, polysaccharide, polyester, and polyamide based polymers according to their chemical composition (Nooeaid et al., 2012; Liu et al., 2018). The natural biological function and ability to promote cell adhesion and proliferation are unique advantages of natural polymer materials such as collagen, gelatin, and chitosan (Nooeaid et al., 2012; Kowalczewski and Saul, 2018). The variability and low mechanical strength of different batches of natural biomaterials lead to inevitable defects (Hsu et al., 2010). In contrast, mechanical properties can be carefully controlled through structural and surface modifications using synthetic polymers (Shimomura et al., 2014). However, because of its inherent hydrophobicity and lack of binding sites, their cell adhesion ability is relatively poor (Sarasam et al., 2006; Antonova et al., 2016). Another important consideration when designing osteochondral scaffolds is that the rate of degradation of biomaterials should match the rate of tissue repair. There are significant differences in the rates at which enzymes degrade natural polymers at different transplant sites *in vivo*, depending on the activity and concentration of the enzyme under different conditions. Conversely, hydrolytically degradable synthetic polymers show minor differences between sites or patients compared to enzymatically degradable polymers. However, the by-products of degradation are toxic (Zhang et al., 2014). Bioceramics, such as calcium phosphate, are characterized by their excellent osteoinductivity. Common types of bone calcium scaffolds are hydroxyapatite, tricalcium phosphate, biphasic calcium phosphate, and multiphase bioglass. By changing the composition of Ca_3_(PO_4_)_2_ ceramics, the stability and mechanical properties of the materials can be modified (Lima et al., 2019). However, separate scaffold biomaterials are not effective in promoting osteochondral tissue repair. To increase the number of cells and the chondrogenesis/osteogenesis of MSCs, an array of cellular factors can be applied to the scaffold to promote and maintain the production of cartilage ECM.

### Biochemical Factors

Bone morphogenetic proteins (BMPs) (Reyes et al., 2014), fibroblast-growth-factor 2 (FGF-2) (Yi et al., 2012), transcriptional SOX proteins (Cao et al., 2011), nel-like molecule-1 (Nell-1) (Zhang et al., 2016b; Wang et al., 2017), and IGF-1 and IGF-2 promote cartilage formation (Wang et al., 2009) and osteogenic differentiation. In addition, as angiogenic factors, Platelet derived growth factors (PDGF), vascular endothelial growth factor (VEGF), and early growth response gene 1 (EGR-1) promote bone repair (Franses et al., 2010; Press et al., 2015; Sheng et al., 2018). As anti-angiogenic factor, chondromodulin 1 (CHM-1) not only stimulates chondrogenesis but also inhibits chondrocyte hypertrophy and endochondral ossification (Klinger et al., 2011). A common route of administration for general growth factors is intravenous injection. However, the growth factor in the blood has a short half-life. By adjusting the physicochemical properties of the scaffold to slow release of growth factors, the drawbacks of direct administration can be avoided. Although 3D scaffolds can function as sustained-release growth factors, their ability to promote perivascular tissue healing and stem cells (SCs) regeneration is limited by their localization. Scaffold-based gene therapy provides a promising approach for tissue engineering through transfecting specific nucleic acids into cells to enhance the sustained expression of the growth factors of interest or through silencing target genes associated with bone and joint disease

### MicroRNAs

#### Cartilage

MicroRNAs (MiRNAs) are ~22 nucleotide single stranded RNAs that regulate post-transcriptional gene expression. MiRNA induces degradation of the target mRNA by binding to the 3′-untranslated region (UTR) complementary sequence on messenger RNA (mRNA), inhibiting translation, thereby suppressing corresponding protein production. Unlike small interfering RNA (siRNA), which regulates only one specific target, miRNA can regulate multiple targets. A single miRNA can regulate different targets in multiple signaling pathways, so it is more advantageous than other biomolecules in terms of functional effects. MiRNA expression profiles are significantly different during the development of articular cartilage, chondrocyte differentiation, and MSC chondrogenesis. Chondrocyte miRNA profiles differ from normal chondrocytes during their degeneration during osteoarthritis (OA). These miRNAs involved in chondrocyte differentiation or degeneration may be used in bioscaffolds in future studies to participate in the regeneration of cartilage tissue. The following is a summary of their latest.

MiRNAs regulate chondrocyte signaling and epigenetic functions (Cong et al., 2017b). Among the miRNAs, miR-210 targets the death receptor-6 (DR6) and inhibits NF-κB signaling in cultured chondrocytes and OA animal models. In addition, by inhibiting karyopherin subunit alpha-3 (KPNA3) gene expression, MiR-26a/MiR-26-b regulate the translocation of NF-κB-p65 to the nucleus (Mirzamohammadi et al., 2014), and their inhibition leads to enhanced COX-2 and MMP-3, -9, -13 expression (Yin et al., 2017). MiR-138 expression is low in OA cartilage compared to normal cartilage whilst p65 is targeted by miR-138 during OA progression (Wei et al., 2017). MiR-27a-3p levels are also lower in OA cartilage (Li et al., 2018a) while miR-139 is highly expressed and inhibits cell viability and migration by inhibiting the expression of EIF4G2 and IGF1R. MiR-139 inhibitors show the opposite effect (Hu et al., 2016).

Through its ability to target FUT1, microRNA-149-5p promotes the proliferation and survival of chondrocytes, thus preventing OA. It has also been found to be downregulated in patients with OA, leading to degenerative cartilage and disturbing homeostasis. Carriers have been employed to deliver miRNA-149-5p to MSCs to promote chondrogenesis (Celik et al., 2019). MiR-218 is highly expressed early in cartilage formation, but is stopped in synaptic-derived mesenchymal stem cells (SDSC) at the maturation stage of cartilage differentiation and miR-218 may directly regulates 15-hydroxyprostaglandin dehydrogenase expression in SDSCs (Chen et al., 2019c). MiR-320c was decreased in the later stages of chondrogenesis of adipose-derived stem cells (hADSCs) and OA chondrocytes. It inhibits degeneration of OA chondrocytes by directly targeting beta-catenin and inhibiting Wnt signaling (Hu et al., 2019). MiR-92a-3p expression was increased in MSC chondrogenic exosomes and significantly decreased in OA chondrocytes exosomes. MiR-92a-3p may be involved in regulating cartilage development by targeting WNT5A (Mao et al., 2018b). Conversely, miR-182-5p plays a negative role in BM-MSC chondrogenesis by down-regulating parathyroid hormone-like hormone (PTHLH) (Bai et al., 2019). **Table 1** summarizes the miRNAs, related to cartilage development, that have been studied in recent years.

**Table 1:** Summary of the miRNAs associated with cartilage development and homeostasis.

miRNA	Targets gene	*In vitro*/*in vivo*	Cells/*vivo* model	Biological effect	Reference
**miR-9-5p**	Tnc	*In vitro*, *in vivo*	Mice chondrocytes, mice	Regulates cartilage homeostasis	(Chen et al., 2019a)
**miR-10a-5p**	HOXA1	*In vitro*, *in vivo*	Mice chondrocytes, mice	Regulates cartilage homeostasis	(Ma et al., 2019b)
**miR-16-5p**	SMAD3	*In vitro*	Human chondrocytes	Regulates cartilage homeostasis	(Li et al., 2015)
**miR-21-5p**	FGF18	*In vitro*, *in vivo*	Human chondrocytes, mice	Regulates cartilage homeostasis	(Wang et al., 2019b)
**miR-27a**	PI3K	*In vitro*	SW1353	Regulates cartilage homeostasis	(Cai et al., 2019)
**miR-30a**	DLL4	*In vitro*	Rat MSC	Enhance chondrogenesis	(Tian et al., 2016)
**miR-34a**	Cyr61	*In vitro*	Human chondrocytes	Regulates cartilage homeostasis	(Yang et al., 2018a)
**miR-92a-3p**	ADAMTS4/5	*In vitro*	Human MSC	Enhance chondrogenesis	(Mao et al., 2017a)
	HDAC2	*In vitro*	Human MSC	Enhance chondrogenesis	(Mao et al., 2017b)
**miR-93**	TCF4	*In vitro*, *in vivo*	Human chondrocytes, rabbit	Regulates cartilage homeostasis	(Xue et al., 2019)
	TLR4	*In vitro*, *in vivo*	Mice chondrocytes, mice	Regulates cartilage homeostasis	(Ding et al., 2019)
**miR-95-5p**	HDAC2/8	*In vitro*	Human chondrocytes	Regulates cartilage homeostasis	(Mao et al., 2018a)
**miR-98**	Bcl-2	*In vivo*	Rats	Regulates cartilage homeostasis	(Wang et al., 2016)
**miR-107**	HMGB-1	*In vitro*, *in vivo*	Human chondrocytes, rabbit	Regulates cartilage homeostasis	(Lin et al., 2019)
**miR-127-5p**	Runx2	*In vitro*	Rat BMSCs	Enhance chondrogenesis	(Xue et al., 2017)
**miR-138**	HIF-2α	*In vitro*	Human chondrocytes	Inhibit chondrogenesis	(Seidl et al., 2016)
**miR-140-5p**	Smad3	*In vitro*	Mandibular condylar chondrocytes	Regulates cartilage homeostasis	(Li et al., 2019c)
**miR-145**	MKK4	*In vitro*, *in vivo*	Rat chondrocytes, rat	Regulates cartilage homeostasis	(Hu et al., 2017)
**miR-145-5p**	SOX9	*In vitro*	Human BMSC	Inhibit chondrogenesis	(Verbus et al., 2017)
**miR−146a−5p**	CXCR4	*In vitro*	Human chondrocytes	Regulates cartilage homeostasis	(Jia et al., 2019)
**miR-146b**	AM	*In vitro*, *in vivo*	Mice chondrocytes, mice	Regulates cartilage homeostasis	(Liu et al., 2019b)
	SOX5	*In vitro*	Human Skeletal stem cells	Inhibit chondrogenesis	(Budd et al., 2017)
**miR-149-5p**	FUT-1	*In vitro*	Human MSC	Enhance chondrogenesis	(Celik et al., 2019)
**miR-181a-5p**	SBP2	*In vitro*	SW1353	Regulates cartilage homeostasis	(Xue et al., 2018)
**miR-193b-3p**	HDAC3	*In vitro*, *in vivo*	hMSC, PHCs, nude mice	Enhance chondrogenesis, Regulates cartilage homeostasis	(Meng et al., 2018)
**miR-221-3p**	SDF1	*In vitro*	SW1353	Regulates cartilage homeostasis	(Zheng et al., 2017)
**miR-222**	HDAC-4	*In vitro*, *in vivo*	Human chondrocytes, mice	Regulates cartilage homeostasis	(Song et al., 2015)
**miR-320**	MMP-13	*In vitro*	Mice chondrocytes	Enhance chondrogenesis	(Meng et al., 2016)
**miR-322**	MEK1	*In vitro*, *in vivo*	Mice chondrocytes, mice	Enhance chondrogenesis	(Bluhm et al., 2017)
**miR-365**	HDAC4	*In vitro*, *in vivo*	Rat BMSCs, rats	Enhance chondrogenesis	(Chen and Wu, 2019)
**miR-384-5p**	SOX9	*In vitro*, *in vivo*	Mice chondrocytes, mice	Regulates cartilage homeostasis	(Zhang et al., 2018)
**miR-410**	Wnt3a	*In vitro*	Human BMSC	Enhance chondrogenesis	(Zhang et al., 2017)
**miR-411**	MMP-13	*In vitro*	Human chondrocytes	Regulates cartilage homeostasis	(Wang et al., 2015)
**miR-483**	SMAD4	*In vitro*	Human BMSC	Enhance chondrogenesis	(Anderson and McAlinden, 2017)
**miR-526b-3p**	SMAD7	*In vitro*	Human BMSC	Enhance chondrogenesis	(Wu et al., 2018)

Tnc, tenascin C; AM, alpha-2-macroglobulin; HMGB-1, high mobility group box 1; CXCR4, C−X−C chemokine receptor type 4; TLR4, toll−like receptor 4; SBP2, sequence binding protein 2; SW1353, human chondrosarcoma chondrocyte; HDAC2/8, histone deacetylase 2/8; hMSC, human mesenchymal stem cell; PHCs, primary human chondrocytes; Cyr61, cysteine-rich angiogenic inducer 61; MKK4, mitogen-activated protein kinase 4; CXCL12, C−X−C motif chemokine ligand 12; MEK1, mitogen-Activated Protein Kinase 1; DLL4, delta-like 4; MMP-13, metalloproteinase 13; HIF-2α, hypoxia-inducible factor 2α.

#### Subchondral Bone

The subchondral bone layer below the cartilage in a joint acts as a shock absorber to absorb stress, cushion vibrations, and maintaining joint shape. Studies have shown that subchondral bone remodeling runs through the entire pathogenesis of OA (Aho et al., 2017) *via* the activities of two main cell populations, osteoblasts (OBs) that promote bone formation and osteoclasts (OCs) that promote bone resorption. OBs originate from MSC precursors mainly through BMPs, Wnt, TGF-β signals. OCs originate from peripheral blood mononuclear cell (PBMC) precursors mainly by the effects of RANKL/OPG ratio. Bone remodeling and osteoclast differentiation are controlled by miRNAs (Taipaleenmaki, 2018). MiR-135-5p promotes osteogenesis through its ability to enhance the activity of alkaline phosphatase (ALP), upregulate calcification molecules, and target the Hypoxia inducible factor 1 alpha inhibitor (HIF1AN) (Yin et al., 2019). Conversely, MiR-145 suppresses human jaw bMSC osteogenic differentiation through WNT/β-catenin signaling and semaphorin3A (SEMA3A) targeting (Jin et al., 2019). Similarly, MiR-494 suppresses osteoblast differentiation by BMPR-SMAD-RUNX2 signal simulated by microgravity (Qin et al., 2019). MiR-877-3p targets Smad7 to enhance TGF-β1 mediated MC3T3-E1 cell differentiation (He et al., 2019). MiR-200c also enhances osteogenic differentiation of hBMSCs by regulating AKT/β-Catenin signaling through the inhibition of myeloid differentiation factor 88 (Myd88) (Xia et al., 2019). In human ADSCs, miR-125a-3p could negatively modulates osteoblastic differentiation *via* targeting Smad4 and Jak1. John et al. found that miR-487b-3p suppressed osteoblast differentiation by targeting Notch-regulated ankyrin-repeat protein (Nrarp), which in turn, suppresses Runx-2 and Wnt signaling (John et al., 2019). In BMSCs, miR-206 inhibits osteogenic differentiation through regulating glutamine metabolism (Chen et al., 2019d). MiR-223 is a newly discovered miRNA that induces MC3T3-E1 differentiation *via* HDAC2 targeting (Chen et al., 2019b). A complete summary is shown in **Table 2**.

**Table 2:** Summary of recently identified miRNAs associated with osteogenesis.

miRNA	Targets gene	*In vitro*/*in vivo*	Cells/*vivo* model	Biological effect	Reference
**miR-16-2-3p**	WNT5A	*In vitro*	hBMSCs	Inhibit osteogenic differentiation	(Duan et al., 2018)
**miR-21-5p**	SMAD7	*In vitro*	MC3T3-E1	Promote osteoblast differentiation	(Li and Jiang, 2019)
**miR-27b**	PPAR	*In vitro*	hBMSCs	Promote osteogenic differentiation	(Seenprachawong et al., 2018)
**miR-29b**	BCL-2	*In vitro*	Mice BMSCs	Promote osteoclast differentiation	(Sul et al., 2019)
**miR-34c**	LGR4	*In vitro*	Mice BMMs	Promote osteoclast differentiation	(Cong et al., 2017a)
**miR-92b-5p**	ICAM-1	*In vitro*, *in vivo*	Mice BMSCs, mice	Promote osteogenic differentiation	(Li et al., 2019d)
**miR-96**	SOST	*In vitro*	Mice osteoblast	Promote osteoblast differentiation	(Ma et al., 2019a)
**miR−100−5p**	FGF21	*In vitro*, *in vivo*	Mice BMMs, mice	Inhibit osteoclast differentiation	(Zhou et al., 2019a)
**miR-125a-5p**	TNFRSF1B	*In vitro*	RAW 264.7 OPC	Promote osteoclast differentiation	(Sun et al., 2019a)
**miR-128**	SIRT6	*In vitro*	C2C12 cells	Inhibit osteoblast differentiation	(Zhao et al., 2019b)
**miR-130a**	PPAR	*In vitro*	hBMSCs	Promote osteogenic differentiation	(Seenprachawong et al., 2018)
**miR-132-3p**	Smad5	*In vitro*	MC3T3-E1	Inhibit osteoblast differentiation	(Liu et al., 2019a)
**miR-135-5p**	HIF1AN	*In vitro*	MC3T3-E1	Promote osteoblast differentiation	(Yin et al., 2019)
**miR-139-3p**	ELK1	*In vitro*	MC3T3-E1	Inhibit osteoblast differentiation	(Wang et al., 2018c)
**miR-140-5p**	TLR4, BMP2	*In vitro*, *in vivo*	ASCs, rats	Promote osteogenesis	(Guo et al., 2019b)
**miR-141**	Calcr, EphA2	*In vitro*, *in vivo*	M-BMMs, monkey	Inhibit osteoclast differentiation	(Yang et al., 2018b)
**miR-142-5p**	PTEN	*In vitro*	Rat BMMs	Promotes osteoclast differentiation	(Lou et al., 2019)
**miR-144-3p**	RANK	*In vitro*	CD14+PBMC	Inhibit osteoclast differentiation	(Wang et al., 2018a)
**miR-145**	SEMA3A	*In vitro*	hJBMMSCs	Inhibit osteoblastic differentiation	(Jin et al., 2019)
**miR-145-5p**	OPG	*In vitro*, *in vivo*	RAW-264.7, mice	Promotes osteoclast differentiation	(Chen et al., 2018)
**miR-146a**	M-CSF	*In vivo*	Mice with OVX	Inhibit osteoblast differentiation	(Zhao et al., 2019a)
**miR-199a-5p**	Mafb	*In vitro*	RAW 264.7 cells	Promote osteoclast differentiation	(Guo et al., 2018)
**miR-218-5p**	COL1A1	*In vitro*	Mice BMSCs	Promote osteoblastic differentiation	(Kou et al., 2019)
**miR-218**	Mmp9	*In vitro*	RAW264.7 cells	Inhibit osteoblastic differentiation	(Guo et al., 2019a)
**miR-200c**	Myd88	*In vitro*	hBMSCs	Promote osteogenic differentiation	(Xia et al., 2019)
**miR-208a-3p**	ACVR1	*In vitro*, *in vivo*	MC3T3-E1, mice	Inhibit osteoblastic differentiation	(Arfat et al., 2018)
**miR-210**	Runx2	*In vitro*	HUCB-MSC	Promote osteoblast differentiation	(Asgharzadeh et al., 2018)
**miR-221**	ZFPM2	*In vitro*	MC3T3-E1	Promote osteoblast differentiation	(Zheng et al., 2018)
**miR−223−5p**	HDAC2	*In vitro*, *in vivo*	MC3T3−E1, mice	Promote osteoblast differentiation	(Chen et al., 2019b)
**miR-338-3p**	IKKβ	*In vitro*	RAW264.7 cell	Inhibit osteoclast differentiation	(Niu et al., 2019)
	RANKL	*In vitro*	Mice BMCs	Inhibit osteoclast differentiation	(Zhang et al., 2016a)
**miR-342-3p**	ATF3	*In vitro*, *in vivo*	MC3T3−E1, mice	Promote osteoblast differentiation	(Han et al., 2018)
**miR-363-3p**	PTEN	*In vitro*	CD14+PBMC	Promote osteoclast differentiation	(Li et al., 2019a)
**miR-367**	PANX3	*In vitro*, *in vivo*	Mice osteoblast, mice	Promote osteoblast differentiation	(Jia and Zhou, 2018)
**miR-376c-3p**	IGF1R	*In vitro*	hBMSCs,	Inhibit osteogenic differentiation	(Camp et al., 2018)
**miR-377**	RANKL	*In vitro*, *in vivo*	hBMMs, mice	Inhibit osteoclast differentiation	(Li et al., 2019b)
**miR-383**	Satb2	*In vitro*	Rat BMSCs	Inhibit osteoblastic differentiation	(Tang et al., 2018)
**miR-494**	BMPR2/RUNX2	*In vitro*	C2C12 cells	Inhibit osteoblast differentiation	(Qin et al., 2019)
**miR-451**	YWHAZ	*In vitro*, *in vivo*	hBMSCs, mice	Inhibit osteoblast differentiation	(Pan et al., 2018)
**miR-487b-3p**	Nrarp	*In vitro*, *in vivo*	Mice osteoblasts, mice	Inhibit osteoblast differentiation	(John et al., 2019)
**miR-874**	SUFU	*In vitro*, *in vivo*	Rat osteoblasts, rat	Promote osteoblast differentiation	(Lin et al., 2018)
**miR-877-3p**	Smad7	*In vitro*	MC3T3-E1	Promote osteoblast differentiation	(He et al., 2019)
**miR-1225**	Keap1	*In vitro*, *in vivo*	BMMs, mice	Inhibit osteoclast differentiation	(Reziwan et al., 2019)
**miR-let-7c**	SCD-1	*In vitro*	hADSCs	Inhibit osteogenic differentiation	(Zhou et al., 2019b)

HIF1AN, hypoxia-inducible factor 1 α inhibitor; M-CSF, macrophage colony-stimulating factor; OVX, ovariectomy; SEMA3A, semaphorin 3A; h-JBMMSCs, human jaw bone marrow mesenchymal stem cells; ICAM-1, intracellular adhesion molecule‐1; SOST, sclerostin; hADSCs, human adipose derived mesenchymal stem cells; Nrarp, notch-regulated ankyrin-repeat protein; PPAR, peroxisome Proliferator-Activated Receptor γ; ATF3, activating transcription factor 3; SCD-1, stearoyl-CoA desaturase 1; ZFPM2, zinc finger protein multitype 2; MC3T3-E1,the mouse osteoblast-like cells; SUFU, suppressor of fused gene; IGF1R, insulin growth factor 1 receptor; HUCB, human umbilical cord blood; Satb2, special AT-rich-sequence-binding protein 2; ACVR1, activin A receptor type I; BMMs, bone marrow-derived macrophages; TNFRSF1B, TNF receptor superfamily member 1B gene; RAW 264.7 OPC, RAW 264.7 osteoclast precursor cell; Mmp9, matrix metalloproteinase-9; OPG, osteoprotegerin; M-BMMs, monkey bone marrow-derived macrophages; Calcr, calcitonin receptors; EphA2, ephrin type-A receptor 2 precursor; LGR4, leucine-rich repeat-containing G-protein-coupled receptor 4.

Similar to osteoblast differentiation, the expression pattern of miRNAs related with the osteoclast differentiation has also been deeply explored (Hrdlicka et al., 2019). MiR-363-3p activated by MYB enhances osteoclast differentiation and inhibits osteoblast differentiation *via* the PI3K-AKT-PTEN axis (Li et al., 2019a). MiR-1225 suppresses TNFα-induced osteoclast differentiation through Keap1-Nrf2-HO-1 signal *via* ROS generation in bone marrow-derived macrophages (BMMs) (Reziwan et al., 2019). Conversely, miR-142-5p targets PTEN and induces BMM osteoclastogenesis (Lou et al., 2019). In addition, Smad3 expression is reduced by miR-145, the mimics of which in OVX mice repress OCs (Yu et al., 2018). MiR-125a-5p promotes osteoclast differentiation through inhibiting TNFRSF1B expression (Sun et al., 2019a). Sun and colleagues showed that miR-338-3p enhances the differentiation of Ocs by targeting Mafb (Sun et al., 2019b) that is also a target for miR-199a-5p (Guo et al., 2018). Wang et al. found that miR-218 decreased osteoclastogenic differentiation *via* suppressing NF-κB signal *via* targeting TNFR1 (Wang et al., 2018b). Recent studies have shown that miR-133a promotes postmenopausal osteoporosis through enhancing OC differentiation (Li et al., 2018b). The culmination of these studies highlight the potential of miRNAs to regulate OC differentiation (**Table 2**).

## Vector Based Gene-Delivery

Tissue-engineering and gene therapy have been used in the treatment of myocardial injuries (Gabisonia et al., 2019), the repair of cartilage defects (Armiento et al., 2018), and the treatment of bone defects (Chen et al., 2019e). Compared with protein-based treatment, gene therapy has two main advantages. Gene therapy is more biologically active and physiological than common recombinant approaches (Raftery et al., 2019). Since the gene fragment itself cannot be efficiently introduced into the cell, an effective vector is required. Gene vectors can be virus-based (lentiviruses or baculoviruses) or non-viral including transfection methods such as lipofectamine, electroporation, and nanoparticles. They all have their own advantages and disadvantages, but in general, the transfection efficiency of current viral vectors is still higher than that of non-viral vectors.

### Viral Vectors

#### Use of Adenoviruses

Adenoviral transgenic efficiency is typically close to 100% *in vitro*. Adenoviruses can transduce different human tissue cells, dividing and non-dividing. The production of high titer adenoviral vectors is simple and no integration into the genomes of human cells occur. As such, adenoviral vectors have been increasingly used in clinical trials of gene therapy and have become the most promising viral vectors, second only to retroviral vectors. In a recent study, it is found that the use of Adenoviral-BMP-2/basic fibroblast growth factor (bFGF)-modified BMMSCs combined with demineralized bone matrix promote bone formation and angiogenesis, successfully repairing canine femoral head necrosis (ONFH) (Peng and Wang, 2017). However, the biggest challenge to the effectiveness of adenoviral approaches are the immune response.

#### Baculovirus Approaches

Baculoviruses show no pathogenicity toward humans and can be used under normal biosafety level 2 conditions. Baculoviruses, like adenoviral, induce both dividing and non-dividing cells. In some recent studies, baculoviruses has been used. Lo and colleagues employed Cre/loxP-based baculovirus vectors in adipose-SCs to enhance bone healing (Lo et al., 2017). Fu and coworkers highlighted the ability of baculoviruses to induce osteogenesis through allogeneic-MSCs (Fu et al., 2015). Despite this promise, the transient expression profiles of baculoviruses limit their use. In an attempt to overcome this issue, Chen and coworkers developed baculoviruses hybridized with the miR-155 scaffolds and the sleeping beauty transposon to sustainably inhibit transgene expression for extended time periods (Chen et al., 2011).

#### Lentiviruses

The advantage of lentiviral vectors are the high levels of foreign gene integration into the host chromosome in cells typically difficult to transfect, including primary cell cultures. Lentiviral vectors can be combined with chondroitin sulfate-hyaluronic acid-silk fibrin composite scaffolds and applied to bone-ligament connections to promote tissue engineering (Sun et al., 2014). In addition, Brunger et al. developed an independent bioactive scaffold that is capable of inducing stem cell differentiation and cartilage ECM formation using lentiviruses (Brunger et al., 2014). Despite the great progress in the study of lentiviral vector, it is still far from clinical application. First, the titer of recombinant virus is still not up to the level of *in vivo* application. Second, due to the complex biological properties of HIV, it is difficult to establish a stable HIV vector like the commonly used mouse retroviral vector, and the established packaging cells are not ideal.

### Non-Viral Gene Delivery Vectors

Commercialized cationic lipids such as Lipofectamine 2000, Lipofectamine 3000, Lipofectamine RNAiMAX, and SiPORT NeoFx are widely used in biomaterial-based gene therapy. In recent studies, using lipofectamine 2000, Anti-miR-221 was transfected into adipose-MSCs which were seeded into synthetic nHA/PCL scaffolds. The results indicate that this method provides an effective way to promote osteogenesis of AT-MSCs (Hoseinzadeh et al., 2016). Macmillan et al. combined lipofectamine-complexed plasmids encoding BMP-2 and TGF-β1 with HA microparticles for delivery to the MSCs of three healthy pig donors. This study provides a promising approach to gene therapy that regulates stem cell growth and development to treat bone defects (McMillan et al., 2018).

Although the toxicity of liposomes are well-known, more efficient transfection methods to replace them have not emerged. Recently, to enhance the interaction between cells and nucleic acids, Raftery et al. developed a new cell penetrating peptide, GET, combined with a variety of collagen scaffolds, which showed good regeneration potential. GET is suitable for all three germ layer cell transfections with efficiencies comparable to Lipofectamine 3000 and minimal cytotoxicity. These findings suggest that GET can be combined with scaffold delivery systems, to provide new solutions to a variety of tissue engineering regenerative indications (Raftery et al., 2019).

## Gene Therapy in Scaffold Based Osteochondral Tissue Repair

**Figure 2:**
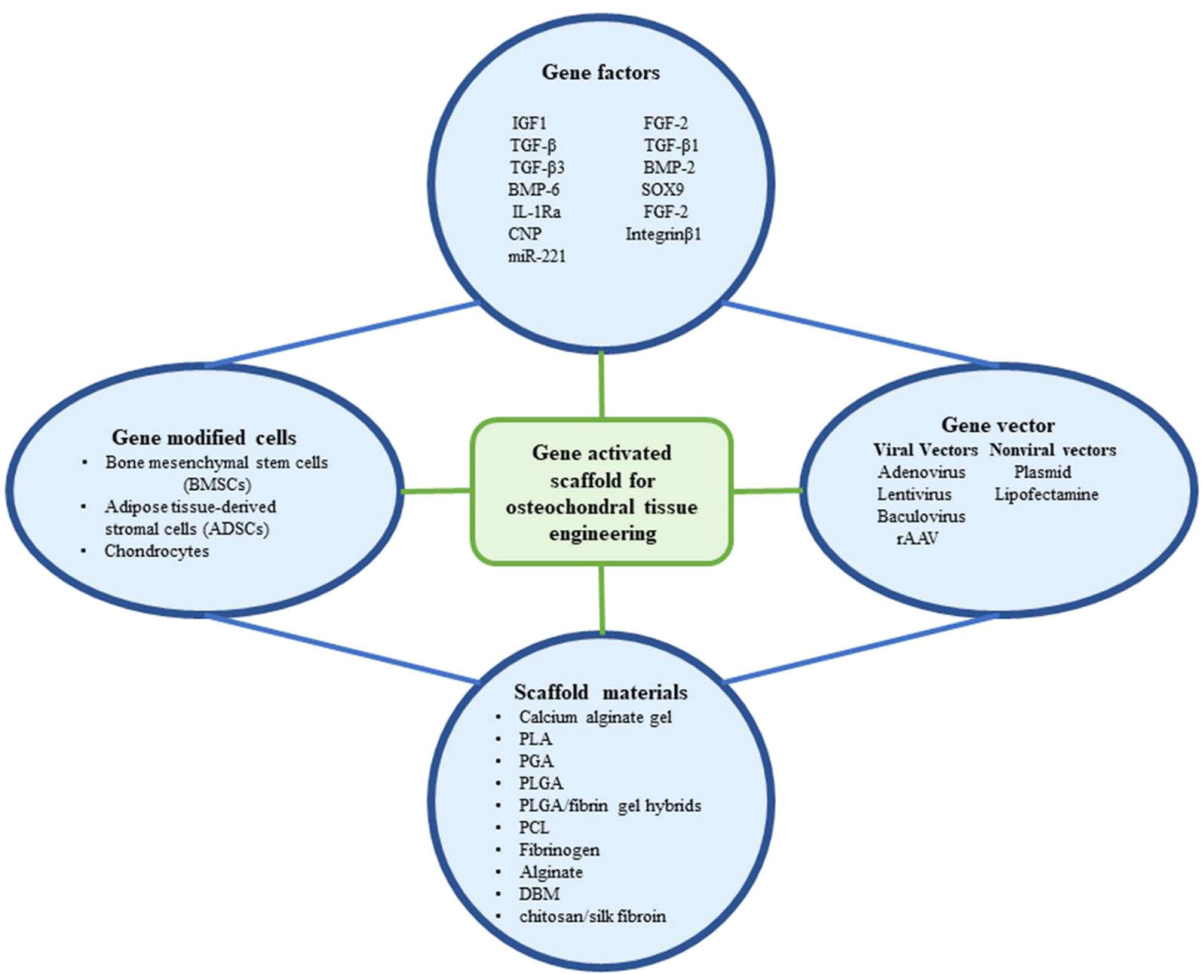
The components that have been utilized in gene activated scaffold for osteochondral tissue engineering.

Gene therapy for osteochondral tissue repair is divided into two phases: one to locate the gene to the target area directly, either through encapsulation onto a scaffold, or through a specific gene vector (*in vivo*). Alternatively, the target gene is loaded into the cells by the vectors *in vitro*, and genetically modified cells are administered to the target lesion area, with or without a scaffold (*ex vivo*). However, the main obstacle to the treatment of focal defects with non-scaffolds is that the genetically modified cells or gene vectors with intra-articular injections are diluted by the joint fluid and fail to reach the target lesion area. To avoid this drawback, a promising approach is to deliver modified cells or gene vectors using different types of scaffolds. When the scaffold is degraded, the contents are slowly released to the target area. Gene therapy combined with scaffolds increases the efficiency and duration of transfected genes, forming an efficient system to promote osteochondral regeneration. We herein summarize and discuss these gene therapy-binding scaffolds discovered from 2006 to 2019 in the contest of seeding cell types (**Figure 2**).

### Gene Modified BMSCs

MSCs are the most widely studied due to their high availability and proliferative/differentiation ability. The microenvironment typically dictates the fate of MSCs. BMSCs are more commonly employed than those derived from adipose tissue (AMSCs), particularly for osteochondral therapy. In one study, BMSCs were transfected with hIGF-1 cDNA and mixed with calcium alginate gels for transplantation into 6 mm osteochondral defects and were found to improve the repair (Leng et al., 2012).

In view of the role of TGF-β in promoting cartilage repair, in addition to the inhibition of inflammatory and immune responses, pcDNA-TGF-β gene-modified BMSCs were seeded onto biodegradable poly-L-lysine coated polylactic acid (PLA) scaffolds which were transplanted into New Zealand rabbit articular cartilage full-thickness defects. *In vitro*, after 2 weeks of cell seeding, the cartilage matrix formed and filled with the attachment holes of the scaffold. *In vivo*, 24 weeks after transplantation, the hyaline cartilage repaired the cartilage defect area, trabecular bone and dense bone repair in the subchondral area and the quality of the regenerated tissue improved over time (Guo et al., 2006). Similarly, BMSCs were transduced with pDNA-TGF-β1 and loaded into PLGA/fibrin gel hybrids which were implanted into rabbit articular cartilage models, resulting in the regeneration of new cartilage tissue with similar thickness, cell arrangements, color, and abundant glycosaminoglycans to normal cartilage (Li et al., 2014). Moreover, TGF-β1-gene engineered rat BMSCs induced cartilage regeneration in rats (He et al., 2012), and their transfer onto PGA scaffolds using adenoviral approaches induced chondrogenic differentiation both *in vitro* and *in vivo* (Xia et al., 2009). Robust collagen II staining was observed in adenovirus-mediated-BMP-2 and TGF-β3 infected cells. DBM compounds with BMP-2 and TGF-β3 infected BMSC scaffolds showed high biocompatibility and the capacity to regeneration cartilage in pig models (Wang et al., 2014).

SOX9 is a transcription factor of the SOX (Sry-type HMG box) protein family that promotes cartilage formation and the phenotypes of chondrocytes. Adenoviral vectors have been used to transfect SOX9 into rabbit BMSCs which effectively induced their differentiation into chondrocytes on PGA scaffolds and improved the repair of cartilage defects (Cao et al., 2011). More recently, Venkatesan et al. designed 3D fibrin-polyurethane scaffolds in a hydrodynamic environment that provided a favorable growth environment for rAAV-infected SOX9-modified hBMCs and promoted their differentiation into chondrocytes. Interestingly, the expression of SOX9 lasted 21 days, the longest time point evaluated (Venkatesan et al., 2018).

Cartilage engineering can also be mediated through gene activation matrices. Rowland et al. engineered lentiviral particles expressing a doxycycline-inducible IL-1 receptor antagonist (IL-1Ra) on a cartilage-derived matrix to prevent IL-1 mediated inflammation. Similar scaffolds have been employed for site directed chondrogenic and osteogenic differentiation using BMSC populations that overexpress either chondrogenic, BMP2, or TGF-β3 transgenes. The ability to regulate IL-1Ra expression afforded protection to the cartilage-matrix in the presence of IL-1, leading to enhanced bone production and cartilage formation. When inflammation is absent, cartilage-derived matrix hemispheres expressing TGF-β3 and BMP-2 were also fused to the bilayers of osteochondral constructs to promote healing (Rowland et al., 2018). Yang and coworkers also transfected BMSCs with adenoviruses expressing C-type natriuretic peptides and seeded the cells onto silk/chitosan scaffolds to promote chondrogenesis in rat cartilage defect models (Yang et al., 2019).

Despite advances in the development of osteochondral repair scaffolds, their combination with miRNAs remains in the early stages. MiR-221 can induce BMSCs toward chondrogenesis in the absence of TGF-β and could repair osteochondral defects following its subcutaneous implantation into mouse models, promoting Collagen type II positive tissue expression that was negative for collagen type X (a well characterized marker of hypertrophy). The potential of hMSCs silenced for miR-221 to suppress collagen type X represents an exciting development with clear therapeutic potential for cartilage repair in the clinic (Lolli et al., 2016).

### Gene Modified ADSCs

It is now well accepted that ADSCs have clinical utility. An advantage is their ability to be collected *via* lipoaspiration, a non-invasive harvesting process. Lu and coworkers developed baculoviruses for FLPo/Frt expression of genetically engineered rabbit ADSCs. The cells were transfected with TGF-β3/BMP-6 and added to PLGA-GCH scaffolds for implantation to cartilage defects in weight-bearing areas, promoting regeneration. The designed neo-cartilages had defined cartilage-specific-structures in the absence of degeneration or hypertrophy (Lu et al., 2014). In other studies, the inguinal fat of rats were transduced with SOX *via* retroviral approaches and ADSCs were collected and seeded into fibrin gels and implanted onto defects in the femur patellar groove. These approaches significantly increased type II collagen expression, GAG levels, and improved cartilage healing (Lee and Im, 2012). Upon seeding the ADSCs into large PCL-scaffolds immobilized with Dox-inducible lentiviruses expressing IL-1Ra, controlled tissue growth and biomimetic cartilage properties were maintained (Moutos et al., 2016).

### Gene Modified Chondrocytes

Isolated cartilage cells can be obtained through enzymatic digestion and can embed into cartilage lacuna, preventing immune cell invasion and organ rejection. However, the cells dedifferentiate overtime and their propensity for cartilage production becomes impaired, limiting their use in clinical application. The use of 3D cultures can mimic the microenvironment of the extracellular matrix permitting the maintenance of phenotypic stability. In this regard, neonatal male foals chondrocytes transduced with IGF-1-adenoviruses and embedded into fibrinogen were implanted into equine defects and conferred high levels of IGF-1 expression and cartilage healing (Goodrich et al., 2007). Griffin and colleagues used a comparable approach with rAAV5 and implanted the carriers into equine femurs, also showing improved graft healing (Griffin et al., 2016). FGF-2 and IGF-I plasmid vectors have also been delivered into Lapine articular chondrocytes. The cells were encapsulated into alginate scaffolds and transplanted onto rabbit knee joint defects for a period of three weeks, in which enhanced IGF-I/FGF-2 levels improved the defects with no adverse effects to the synovial membrane, highlighting the utility of these approaches to promote cartilage repair (Orth et al., 2011).

FGF-2 is mitogenic in articular chondrocytes and when transfected into articular chondrocytes and encapsulated in alginate scaffolds, FGF-2 expression was maintained for over 21 days and improved cartilage defects in the knee joints of rabbits. No adverse effects were again evident in the synovial membrane following histological assessments but type II collagen expression was enhanced (Kaul et al., 2006).

Mechanical movements activate integrin β1-signaling and enhance the proliferative capacity of chondrocytes, increasing matrix synthesis. Liang and co-workers seeded integrin β1-transfected chondrocytes onto PLGA scaffolds which produced higher levels of GAG and type II collagen after lentiviral-integrin β1 transfection compared to mechanically stressed sham controls. The opposing phenotype was observed in the cells silenced for integrin β1, suggesting that in addition to mechanical stimulation, the overexpression of integrin β1 enhances cartilage regeneration (Liang et al., 2015) (**Table 3**).

**Table 3:** Summary of gene therapy in scaffold based osteochondral tissue repair.

Cells	Gene	Scaffold	Gene vector	Approach	*Vitro* or *vivo* model	Reference
**BMSCs**	IGF1	Calcium alginate gel	Plasmid	*Ex vivo*	Rabbit knee osteochondral defect	(Leng et al., 2012)
	TGF-β	PLA	Plasmid	*Ex vivo*	Rabbit knee full-thickness defects	(Guo et al., 2006)
	TGF-β1	PLGA/fibrin gel hybrids	Plasmid	*Ex vivo*	Rabbit knee full-thickness defects	(Li et al., 2014)
	TGF-β1	PGA	Adenovirus	*In vitro*, *ex vivo*	Mice subcutaneous tissue	(Xia et al., 2009)
	BMP-2, TGF-β3	DBM	Adenovirus	*In vitro*, *ex vivo*	Pig knee full-thickness defects	(Wang et al., 2014)
	SOX9	PGA	Adenovirus	*In vitro*, *ex vivo*	Rabbit knee full-thickness defects	(Cao et al., 2011)
	SOX9	Fibrin-polyurethane	rAAV	*In vitro*	Hydrodynamic culture conditions	(Venkatesan et al., 2018)
	IL-1Ra, BMP-2, TGF-β3	CDM	Lentiviral	*In vitro*	Joint organoid model	(Rowland et al., 2018)
	CNP	Chitosan/silk fibroin	Adenovirus	*Ex vivo*	Rat knee full-thickness defects	(Yang et al., 2019)
	miR-221	Alginate	Lipofectamine	*In vitro*, *ex vivo*	Mice knee osteochondral defects	(Lolli et al., 2016)
**ADSCs**	TGF-β3/BMP-6	PLGA-GCH	Baculovirus	*Ex vivo*	Rat knee full-thickness defects	(Lu et al., 2014)
	SOX trio	Fibrin gel	Retrovirus	*In vitro*, *ex vivo*	Rat knee osteochondral defect, OA	(Lee and Im, 2012)
	eGFP, IL-1Ra	PCL	Lentiviral	*In vitro*	Cultured in chondrogenic conditions	(Moutos et al., 2016)
**Chondrocytes**	IGF-1	Fibrinogen	Adenovirus	*Ex vivo*	Equine knee osteochondral defect	(Goodrich et al., 2007)
	IGF-1	Fibrin	rAAV5	*Ex vivo*	Equine knee full-thickness defects	(Griffin et al., 2016)
	IGF-I, FGF-2	Alginate	Plasmid	*Ex vivo*	Rabbit knee osteochondral defect	(Orth et al., 2011)
	FGF-2	Alginate	Plasmid	*In vitro*, *ex vivo*	Rabbit knee osteochondral defect	(Kaul et al., 2006)
	Integrin β1	PLGA	Lentiviral	*In vitro*	Cultured under periodic mechanical stress	(Liang et al., 2015)

IGF, Insulin-like growth factor; TGF, Transforming growth factor; BMP, Bone morphogenetic protein; PLGA, Poly lactide-co-glycolide; DBM, Demineralized bone matrix; PGA, polyglycolic; GCH, gelatin, chondroitin-6-sulfate and hyaluronic acid; IL-1Ra, IL-1 receptor antagonist; eGFP, enhanced green fluorescent protein.

## Conclusion and Future Directions

In summary, osteochondral defects are not a single cartilage or bone injury, but involve complex multi-structural components. The healing of these components is challenging. To-date, there is no technology that can form a natural cartilage structures in the joints. Osteochondral tissue engineering shows good potential for osteochondral repair and OA treatment, but several problems remain. For example, at the seed cell level, chondrocytes have poor availability and dedifferentiation properties. Unacceptable outcomes such as chondrocyte hypertrophy and endochondral ossification are often accompanied by an inability to control the differentiation of chondrogenic SCs. Also, due to its unique layered structure, osteochondral tissue theoretically requires a multi-phase structure to simulate the native layered structure, but this is difficult to achieve. Recent studies have shown that a combination of gene vectors, genes, seed cells, and scaffolds are more likely to obtain hyaline cartilage, with the combined changes between them primarily based on lesion size, location, and structure.

Genes have been transfected into MSCs or chondrocytes to improve their phenotypic properties. In general, cartilage gene therapy enables seed cells to continuously encode growth factors, transcription factors, or anti-inflammatory cytokines, thereby inducing cartilage differentiation and inhibiting the progression of inflammatory diseases (**Figure 2**). Studies have shown that multiple combinations of genes encoding growth factors, transcription factors, or anti-adverse response cytokines are more advantageous than single genes for improving healing and reducing adverse effects. To minimize hypertrophy, ossification, and host immune responses, complex gene delivery vectors must be designed to increase safety and more sustained gene protein release. miRNAs regulate chondrogenesis and arthritis. The expression of a specific miRNA mimetic or miRNA inhibitor permits the manipulation of the expression profiles of the cellular miRNAs and their epigenetic features. On this basis, combined with 3D biological scaffold printing technology, it is more conducive to accurately control cell differentiation and optimize the biochemical and biomechanical properties of regenerated tissues. However, the use of 3D delivery systems to miRNA-activated scaffolds is in its infancy. Moreover, in terms of scaffolds, 3D multiphase structural scaffolds are complex, and not conducive to the control of each phase, including degradation rates and shear forces. Therefore, the two-phase scaffold divided into a cartilage phase and a bone phase is simpler than multi-phase scaffolds and ideal for osteochondral scaffolds (Seo et al., 2014). We propose that to make full use of the integrated fusion bilayer scaffold, each genetically modified cell line (overexpression or knockout of miRNA) can edit specific signaling molecules that facilitate tissue regeneration in each layer.

## Author Contributions

XY, Y-RC, and Y-FS proposed and wrote the manuscript. MY, JY, and GZ collected and analyzed the information. J-KY supervised the conception and writing of the manuscript.

## Funding

The research was supported by the National Natural Science Foundation of China (Grant Nos. 51773004, 81630056, 51920105006, 51803188, 31670982) and the National Key Research and Development Program (Grant No. 2016YFC1100704).

## Conflict of Interest

The authors declare that the research was conducted in the absence of any commercial or financial relationships that could be construed as a potential conflict of interest.
